# Prognostic analysis of percutaneous vertebroplasty (PVP) combined with ^125^I implantation on lumbosacral vertebral osteoblastic metastases

**DOI:** 10.1186/s12957-023-03268-3

**Published:** 2023-12-20

**Authors:** Lei Xu, Xin Huang, Yan Lou, Wei Xie, Jun He, Zuozhang Yang, Yihao Yang, Ya Zhang

**Affiliations:** 1 Department of Orthopedics, Chengdu Seventh People’s Hospital (Affiliated Cancer Hospital of Chengdu Medical College), Chengdu, Sichuan 610213 China; 2 Department of Orthopedic Oncology, Spine Tumor Center, Changzheng Hospital, Naval Military Medical University, 415 Fengyang Road, Shanghai, 200003 China; 3 Bone and Soft Tissue Tumors Research Center of Yunnan Province, Department of Orthopaedics, The Third Affiliated Hospital of Kunming Medical University, Yunnan Cancer Hospital, Yunnan Cancer Center, Kunming, Yunnan 650118 People’s Republic of China

**Keywords:** Lumbosacral vertebral metastases, Osteoblastic, Percutaneous vertebroplasty, ^125^I seeds, Prognostic analysis

## Abstract

**Objective:**

Lumbosacral vertebral osteoblastic metastasis is treated with percutaneous vertebroplasty (PVP) combined with ^125^I seed implantation and PVP alone. Compared to PVP alone, we evaluated the effects of combination therapy with PVP and ^125^I seed implantation on pain, physical condition, and survival and evaluated the clinical value of PVP combined with ^125^I particle implantation.

**Methods:**

We retrospectively analyzed 62 patients with lumbosacral vertebral osseous metastases treated at our hospital between 2016 and 2019. All the patients met the inclusion criteria for ^125^I implantation, and they were randomly divided into a combined treatment group and a pure PVP surgery group. The visual analog pain scale (VAS), Karnofsky Performance Status (KPS), and survival time were recorded at different time points, including preoperative, postoperative 1 day, 1 month, 3 months, 6 months, 12 months, and 36 months in each group. The variation in clinical indicators and differences between the groups were analyzed using SPSS version 20.0. Correlations between different variables were analyzed using the nonparametric Spearman’s rank test. The Kaplan–Meier method was used to estimate the relationship between survival time and KPS score, VAS score, or primary tumor progression, and survival differences were analyzed using the log-rank test. Multivariate analyses were performed using a stepwise Cox proportional hazards model to identify independent prognostic factors.

**Results:**

Compared to the PVP treatment group, the pain level in the combined treatment group was significantly reduced (*P* = 0.000), and the patient’s physical condition in the combination treatment group significantly improved. Kaplan–Meier analysis showed that the survival rate of the PVP group was significantly lower than that of the combination group (*P* = 0.038). We also found that the median survival of patients in both groups significantly increased with an increase in the KPS score (14 months vs. 33 months) (*P* = 0.020). Patients with more than three transfer sections had significantly lower survival rates than those with one or two segments of the section (*P* = 0.001). Further, Cox regression analysis showed that age (*P* = 0.002), the spinal segment for spinal metastasis (*P* = 0.000), and primary tumor growth rate (*P* = 0.005) were independent factors that affected the long-term survival of patients with lumbosacral vertebral osseous metastases.

**Conclusions:**

PVP combined ^125^I seeds implantation surgery demonstrated superior effectiveness compared to PVP surgery alone in treating lumbosacral vertebral osseous metastases, which had feasibility in the clinical operation. Preoperative KPS score, spine transfer section, and primary tumor growth rate were closely related to the survival of patients with lumbosacral vertebral osteoblastic metastasis. Age, spinal segment for spinal metastasis, and primary tumor growth can serve as prognostic indicators and guide clinical treatment.

## Introduction

Spinal metastases, including osteolytic, osteoblastic, and mixed bone metastases, are common complications of malignant cancers [1, 2]. Osteoblastic metastases occur most commonly in patients with prostate [3–6], gastric  [7, 8], bladder [9, 10], lung [11, 12], and breast cancer [13–15]. The incidence of metastatic spinal tumors has gradually increased recently with the increasing prevalence of various tumors worldwide [16]. Pain is the main symptom of osteoblastic spinal metastasis. In severe cases, it can cause complications, such as pathological fractures of the vertebral body, compression of the spinal cord, and paraplegia [17]. Therefore, early diagnosis and appropriate treatment are crucial. It is helpful to minimize bone destruction of the vertebral body caused by spinal metastases, restore the physiological function of the patient’s spine as much as possible, and improve the quality of life [18].

In recent years, percutaneous vertebroplasty (PVP) has become a surgical method for the treatment of osteolytic destruction of bone metastases [19–23]. PVP surgery for osteoblastic metastases is rare [21]. Owing to the high rigidity of osteoblastic metastases, the puncture path is hindered [24]. Therefore, spinal osteoblastic metastasis is normally considered a contraindication for PVP [21, 25, 26]. Yang et al. found that the tumor itself and its surrounding areas are the target areas for PVP treatment [27]. Injecting bone cement into these areas can cover the tumor foci, kill them, and reinforce the mechanical strength around the tumor, thereby relieving pain and preventing further destruction of the vertebral body.

^125^I particles have a long half-life, low energy, persistence, and precise positioning. They target the uncontrolled proliferation of tumor tissue cells and have a tumor-killing effect [28, 29, 21, 30]. The radioactive source is accurately implanted into the target tissue, and the source is reasonably distributed according to the volume, density (half-valent layer) of the target tissue, and relationship with the neighboring important organs. This approach enables ‘directed blasting’ and maximizes the destruction of cancer cells while minimizing damage to normal tissues and functions. Inspired by the concept of compound technology, Zuozhang et al. [31] first proposed a combination of PVP and ^125^I seed vertebral body implantation interstitial brachytherapy (Interstitial Brachytherapy) for the treatment of spinal metastases, with a significant curative effect. Armstrong et al. used ^125^I seeds to permanently implant 35 patients with paravertebral tumors, and the local control rate at 1 year was 51% [32]. The implantation of ^125^I seeds in the surgical area can effectively prevent local recurrence after the palliative resection of non-small cell lung cancer. Moreover, 56 patients with liver metastases from colorectal cancer underwent ^125^I seed permanent implantation. The local control rates at 1, 3, and 5 years were 41%, 23%, and 23%, respectively [33, 34]. All the above results show that the permanent implantation of ^125^I seeds can better inhibit tumor growth, control local tumor development, significantly reduce the tumor recurrence rate in situ, relieve pain, and significantly prolong survival.

There have been studies on spinal osteoblastic metastases in the thoracic segment; however, few studies have been conducted on the lumbosacral segments [35].

This study retrospectively analyzed the effects of clinical treatment in 62 patients with lumbosacral spinal metastases at our hospital. The correlation between surgical methods and postoperative survival rates was compared. Additionally, we compared the factors affecting the survival of patients with spinal lumbosacral osteoblastic metastases and the selection and efficacy of various surgical procedures.

## Material and Methods

### Clinical data collection

The subjects of this study were inpatients in the Department of Orthopedics, Chengdu Seventh People’s Hospital (Affiliated Cancer Hospital of Chengdu Medical College), between February 2016 and June 2019.

The inclusion criteria were (a) clinical diagnosis of spinal metastases with a pathological diagnosis of the primary tumor or diseased vertebral body, (b) osteogenetic destruction of the lumbosacral vertebrae, (c) Low back pain is the main clinical manifestation, (d) expected survival time > 3 months, (e) Frankel classification of spinal cord function evaluation grade D, Karnofsky Performance Status (KPS) score > 60 points, (f) systemic conditions permit the procedure; no serious diseases of the heart and brain and other important organs, and ability to lie in the prone position for 1–2 h (g) persistent pain, no significant improvement with drugs, physical therapy, among others. (h) All patients received conventional chemotherapy and other comprehensive treatments according to the primary tumor regimen after the operation.

The exclusion criteria were as follows: (a) diagnosed as primary malignant tumor of the spine, (b) refused or unable to cooperate with the completion of clinical data collection or follow-up because of objective reasons, (c) patients or their family members refuse surgical treatment, (d) KPS score ≤ 40 or combined with heart, lung and other organ failure and unable to tolerate surgery.

### Surgical materials and instruments

- Bone cement injection molding instruments and medicines

**Fig. 1:**
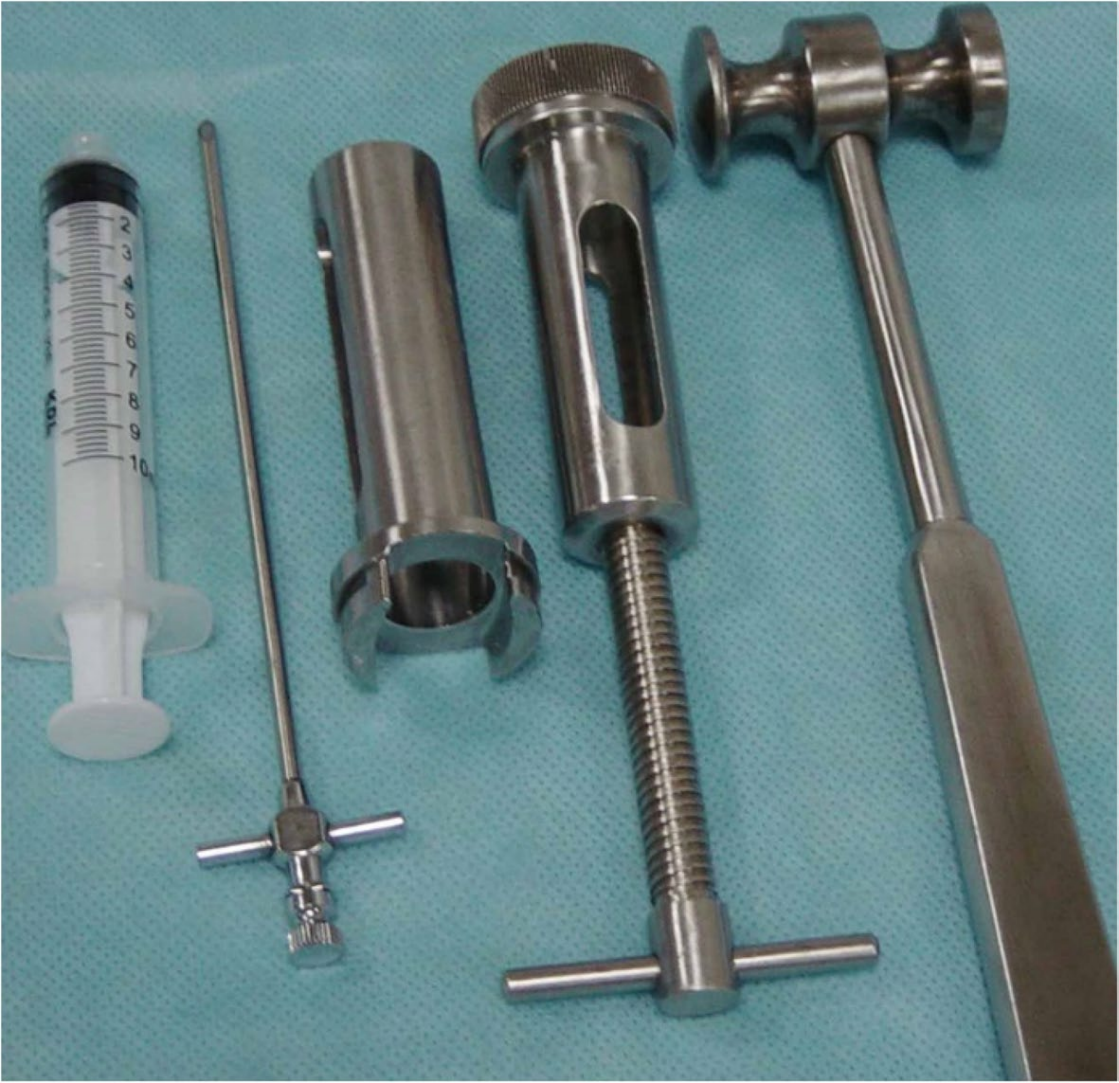
Percutaneous vertebroplasty devices

DOMEStic PVP instruments, including a puncture needle and screw-in syringe pressure device (Shandong Guanlong Company) (Fig. 1). Bone cement: polymethylmethacrylate (PMMA) monomer and polymer type II bone cement were produced by the Tianjin Institute of Synthetic Materials Industry.

(b) Radioactive source

The closed radioactive isotope ^125^I source was provided by Isotope of China Institute of Atomic Energy (National Medicine Zhunzi H20045969). The radioactive source is cylindrical, and its diameter and height are 0.8 mm and 4.5 mm, respectively. The tissue penetration ability is 1.7 cm, the semivalent layer is 0.025 mmPb, the surface is wrapped in titanium alloy, and the source activity is 0.3–1 mCi. A liquid immersion disinfection method was used, and the benzalkonium bromide (Xinjieerfen) solution was soaked for 30 min.

(c) Contrast agent

We used 76% compound meglumine injection, packaged in 20 mL/15.2 g, with an iodine content of 370 mg/mL as a contrast agent (Schering (Guangzhou) Pharmaceutical Co., Ltd., China).

### Research methods

The injection doses of bone cement and I ions, as well as the needle insertion coordinates and depth indications of the applicator, were calculated using the treatment planning system (TPS) software. Specifically, routine preoperative examinations included routine blood tests, blood type, coagulation, blood sugar, liver and kidney function, electrocardiogram, chest radiography, anterior and lateral radiographs of the diseased vertebra, computed tomography (CT), magnetic resonance imaging (MRI), and whole-body bone scans. If conditions permit, a positron emission tomography-computed tomography (PET-CT) examination was performed. Before surgery, CT/MRI images of each patient were scanned into the TPS software for three-dimensional digital image reconstruction. Three-dimensional icons, isodose curves, and absorbed dose indications were accurately formulated and drawn based on the size, location, and relationship with the surrounding normal tissues of the lesion. At the same time, the initial dose of the radiation source required for clinical use, needle insertion coordinates, and depth indications of the applicator are determined, and the treatment plan table is printed out.

The treatment plan was determined based on the tumor source, lesion segment, spinal stability, and degree of intraspinal compression after discussion in the department, and an individualized comprehensive treatment plan was determined. The patients and their families were informed of the treatment plan, and informed consent was obtained from all patients.

### Patient grouping

The patients treated with PVP combined with ^125^I seed implantation were the study group, while the patients treated with PVP alone were the control group.

### Clinical data collection

We explained the purpose and significance of the clinical investigation to the patient, solicited their cooperation and trust, determined the initial visual analog pain scale (VAS) and KPS scores, and recorded the results.

### Postoperative treatment

Postoperative CT scans were used to observe the distribution of bone cement and particles in the vertebral body, whether there was bone cement leakage, among others; observe the symptoms and signs of the patient, whether there was an infection, among others; and provide preventive anti-inflammatory, hemostasis, dehydration, etc., and nutritional nerve processing.

#### Postoperative follow-up

The patients were instructed to retest the VAS before surgery and at 1 month, 3 months, 6 months, 1 year, and 3 years after surgery to evaluate recovery. The KPS was monitored before surgery and at 1 month after surgery.

### Statistical methods

All data were analyzed using SPSS version 20.0 software (IBM Corp., Armonk, N.Y., USA). A *P* value < 0.05 was considered statistically significant. All patients were randomly divided into two groups, and the VAS score was determined before surgery and 1 month, 3 months, 6 months, and 1 year after surgery. Repeated measures analysis of variance was used to determine whether there was a statistical difference in pain between patients before and after surgery, and the scientific significance and effects of different surgical methods on pain in patients.

All patients in this group were scored using the KPS scoring system before and 1 month after the operation, and a *t*-test was used to determine whether the difference in physical strength between the preoperative and postoperative patients was statistically significant.

Univariate analysis (Kaplan–Meier analysis) was conducted. Patients were divided into groups according to sex, age, location, KPS score, VAS score, primary tumor growth, organ metastasis, and surgical method. Univariate survival analysis was performed between the groups and the Kaplan–Meier method was used to analyze the survival time of patients and determine whether each factor affected survival time.

For each prognostic factor (sex, age, location, KPS score, VAS score, primary tumor growth, organ metastasis, and surgical method), a multivariate Cox proportional hazards regression model was used.

## Results

### Basic information on the selected patients

In total, 62 patients with lumbosacral bone metastases were randomly divided into a PVP combined with ^125^I seed implantation group (combined treatment group, *n* = 31) and a PVP alone group (*n* = 31). The clinical data are presented in Table 1, and there was no difference in the factors (all *P* > 0.05). Both groups received conventional chemotherapy according to the primary disease status after surgery.

**Table 1:** Preclinical data analysis of 62 cases of osteoblastic metastasis in patients with lumbosacral vertebral

Factors	PVP group	Combination therapy group	*P* value
age	61.35 ± 3.04	60.87 ± 3.68	0.574
Gender			
Male	17	19	0.614
Female	14	12	
Primary tumor			
Prostate cancer	14	15	0.809
Lung cancer	8	7	
Breast cancer	7	8	
Bladder cancer	1	0	
Duodenal papillary adenocarcinoma	1	1	
Metastatic site			
Lumbar spine	10	11	0.878
Sacral spine	9	12	
Lumbar and sacral spine	12	8	
Spinal segment			
1 segment	8	10	0.211
2 segments	10	12	
≥ 3 segments	13	9	
VAS score	8.71 ± 0.34	8.68 ± 0.33	0.734
KPS score	65.29 ± 4.59	67.71 ± 5.89	0.076

### Evaluation of clinical efficacy

All patients in the combined treatment group were followed up after surgery, and the patients and their families cooperated to complete the evaluation of various efficacy indicators. At the end of the study, 14 of the 31 patients survived. Similarly, all patients in the pure PVP group were followed up after surgery, and they were willing to cooperate in evaluating various efficacy indicators. At the end of the study, five of the 31 patients survived.

### Evaluation of treatment effect

**Fig. 2:**
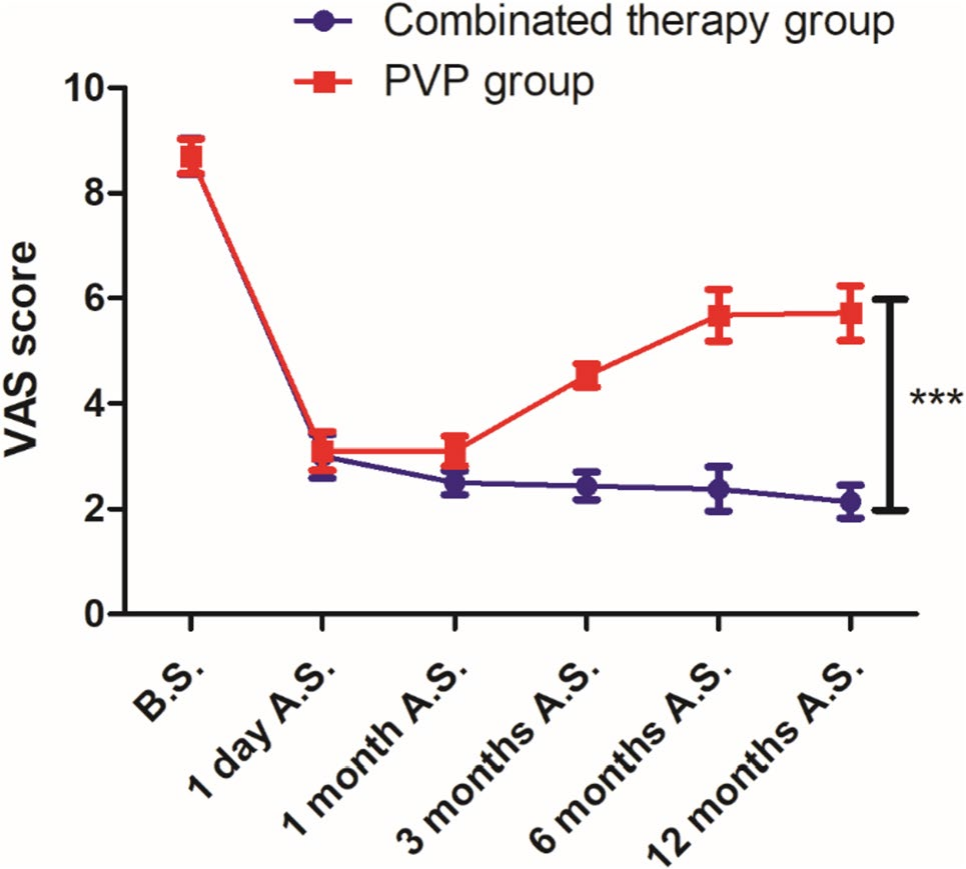
Pain level trends of different surgical methods

We conducted intragroup statistical analyses at different time points for the two groups. As shown in Table 2, the VAS scores of both groups were significantly reduced on the 1st day after the operation. The pain levels in the combined treatment group remained unchanged from 1 to 12 months after surgery. The pain was effectively relieved, while the pain in the simple treatment group was relieved 1 d and 1 month after the operation; however, the pain level gradually increased 3 months after the operation and then remained at a certain level (*P* = 0.000). There was a statistically significant difference in the VAS score between preoperative and postoperative patients. Further, we compared between groups, and the pain level in the combined treatment group was significantly lower than that in the PVP treatment group alone (*P* = 0.000) (Fig. 2).

**Table 2:** VAS score comparisons for the pain of different surgical methods ($\overline{x}$ ± s)

Groups	Cases	Before surgery	After surgery				
			Day 1	1 month	3 months	6 months	12 months
Combination therapy group	31	8.7 ± 0.34	3.0 ± 0.41	2.5 ± 0.23	2.44 ± 0.26	2.38 ± 0.42	2.14 ± 0.31*^△^
PVP group	31	8.7 ± 0.33	3.1 ± 0.36	3.1 ± 0.28	4.53 ± 0.22	5.68 ± 0.49	5.72 ± 0.52^△^

^△^*P* = 0.000 compared with the preoperative and the postoperative

^*^P = 0.000, PVP group compared with combination group

**Fig. 3:**
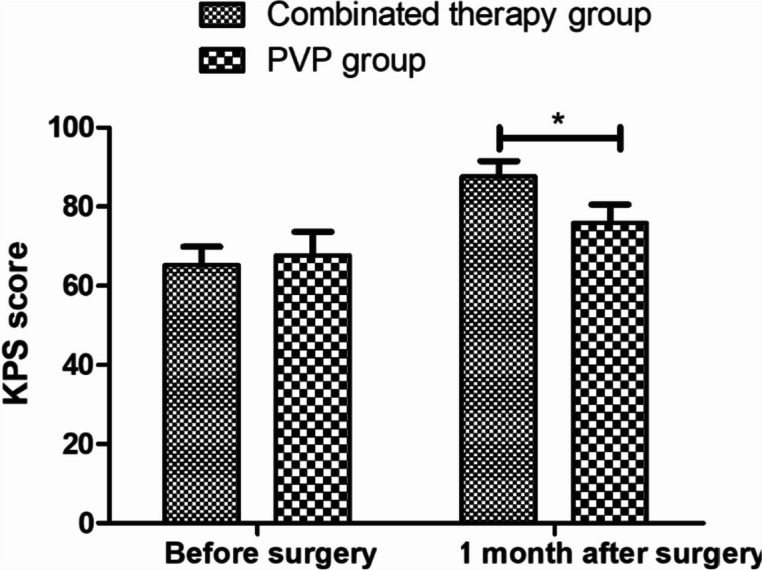
KPS score changes before and after surgery

We statistically analyzed the preoperative KPS scores of the combined treatment group and the pure PVP group, and the results showed that there was no statistical difference between the two groups (65.3 ± 4.6, 67.7 ± 5.9, *P* = 0.076). Additionally, we performed intragroup statistics on the two groups before and 1 month after surgery. The results showed that the KPS scores of the two groups significantly increased 1 month after surgery, indicating that the physical condition of the patients 1 month after surgery was improved compared with that before the operation (Table 3). We further compared the two groups before and 1 month after surgery and found a significant difference between them (*P* = 0.000, Fig. 3).

**Table 3:** Compare KPS score in two groups after treatment ($\overline{x}$ ± s)

Groups	Cases	Before surgery	1 month after surgery
Combined treatment	31	65.3 ± 4.6	87.6 ± 3.9
PVP only treatment	31	67.7 ± 5.9	77.9 ± 4.6
*P*		> 0.05	< 0.05

### Univariate analysis

#### Kaplan–Meier analysis

**Fig. 4:**
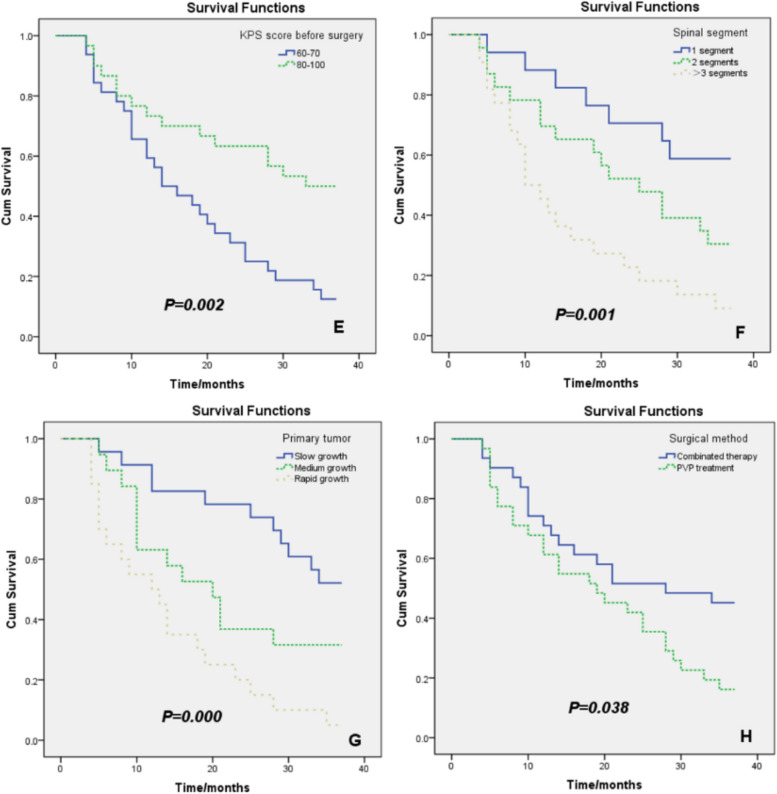
Kaplan–Meier analysis and the log-rank test comparing the influence of factors, such as sex, age, tumor metastases, viscera metastasis, KPS score, spine transfer section, and primary tumor growth, surgical approach on patients’ survival

The log-rank method was used to compare the effects of sex, age, location, KPS score, spinal segment, primary tumor growth, organ metastasis, and surgical method on the survival rate of patients. Survival curves of the survival rates are shown in Table 4 and Fig. 4.

**Table 4:** Survival time for 62 cases of lumbosacral vertebral osteoblastic metastasis

Groups	*n*	Survival rate (%)			Median time (months)	95%CI
		6 months	1 year	3 years		
Surgical approach						
Combination therapy group	31	0.839	0.613	0.452	28	-
PVP alone	31	0.839	0.613	0.161	19	9.002 ~ 28.998
KPS score before surgery						
60–70	30	0.813	0.656	0.125	14	7.070 ~ 20.930
80–100	30	0.867	0.733	0.500	33	14.252 ~ 27.748
Primaries						
Slow growth	23	0.957	0.826	0.522	-	-
Moderate growth	19	0.895	0.632	0.316	20	15.653 ~ 26.558
Rapid growth	20	0.650	0.500	0.050	12	9.932 ~ 18.868
Vertebral segments						
1 segment	17	0.941	0.882	0.588	-	-
2 segments	23	0.826	0.696	0.304	25	18.117 ~ 28.231
≥ 3 segments	22	0.773	0.445	0.091	10	10.986 ~ 19.923

The results showed that there was no significant difference in the survival rate between male and female patients with lumbosacral osteoblastic metastases (*P* = 0.880). The difference in survival rate was not statistically significant between patients aged ≤ 60 years and > 60 years old (*P* = 0.689). Additionally, there was no statistically significant difference in the survival rates between patients with metastatic sites (*P* = 0.967) and those with lumbosacral bone metastases (*P* = 0.092) (Fig. 4).

The survival rate of patients with a preoperative KPS score of 60–70 was significantly lower than that of patients with a score of 80–100 (*P* = 0.002). A higher KPS score indicated a better prognosis. The survival rate of patients with more than three metastatic segments was significantly lower than that of patients with lumbosacral osteoblastic metastases in one and two segments (*P* = 0.001). The survival rate of patients with rapidly growing tumors was significantly lower than that of patients with moderate and slow growth (*P* = 0.000), indicating a low degree of malignancy, slow growth, and long survival time. In contrast, the primary tumor was malignant with high severity, rapid disease progression, and short survival time.

To determine the effects of different surgical methods on the progression-free survival rate of patients with lumbosacral osteoblastic metastases, we used the Kaplan–Meier log-rank method for comparison. The survival curves comparing the different surgical modalities are shown in Fig. 4. In the overall comparison, the survival rate of the PVP group was significantly lower than that of the combined treatment group (*P* = 0.038).

#### Cox regression multivariate analysis

As a multivariate survival analysis model, the Cox regression model can effectively process the final examination data, control various confounding factors, quantitatively analyze the effect strength and direction of the observation indicators, and comprehensively analyze the effect of prognostic factors. To further determine the factors affecting the survival rate of patients with lumbosacral osteoblastic metastases, we analyzed all covariates using the Cox regression equation.

The patient’s sex, age, location, preoperative KPS score, spinal segment, primary tumor growth, organ metastasis, and surgical method were included in the multivariate Cox proportional hazards regression model. The variables and their respective values that finally contributed to the effect equation are presented in Table 5.

**Table 5:** Sixty-two cases of lumbosacral vertebral osteoblastic metastasis multivariate Cox regression analysis

	*B*	SE	Wald	Sig	Exp(B)	95.0%CI for Exp(B)
Age	1.154	0.380	9.210	0.002	3.170	1.505 ~ 6.680
Primary tumor	0.832	0.238	12.266	0.000	2.298	1.443 ~ 3.662
Spinal segment	0.647	0.230	7.873	0.005	1.909	1.215 ~ 2.999

Cox regression analysis showed that age, primary tumor, and metastatic spinal segment were independent predictors of long-term survival in patients with lumbosacral osteoblastic metastases.

#### Relative risk (RR value)

For each level of age, the risk of death increased by 3.170 times. Similarly, for each level of the spinal segment, the risk of death increased by 1.909 times, and for each level of primary tumor nature, the risk of death increased by 2.298 times.

**Fig. 5:**
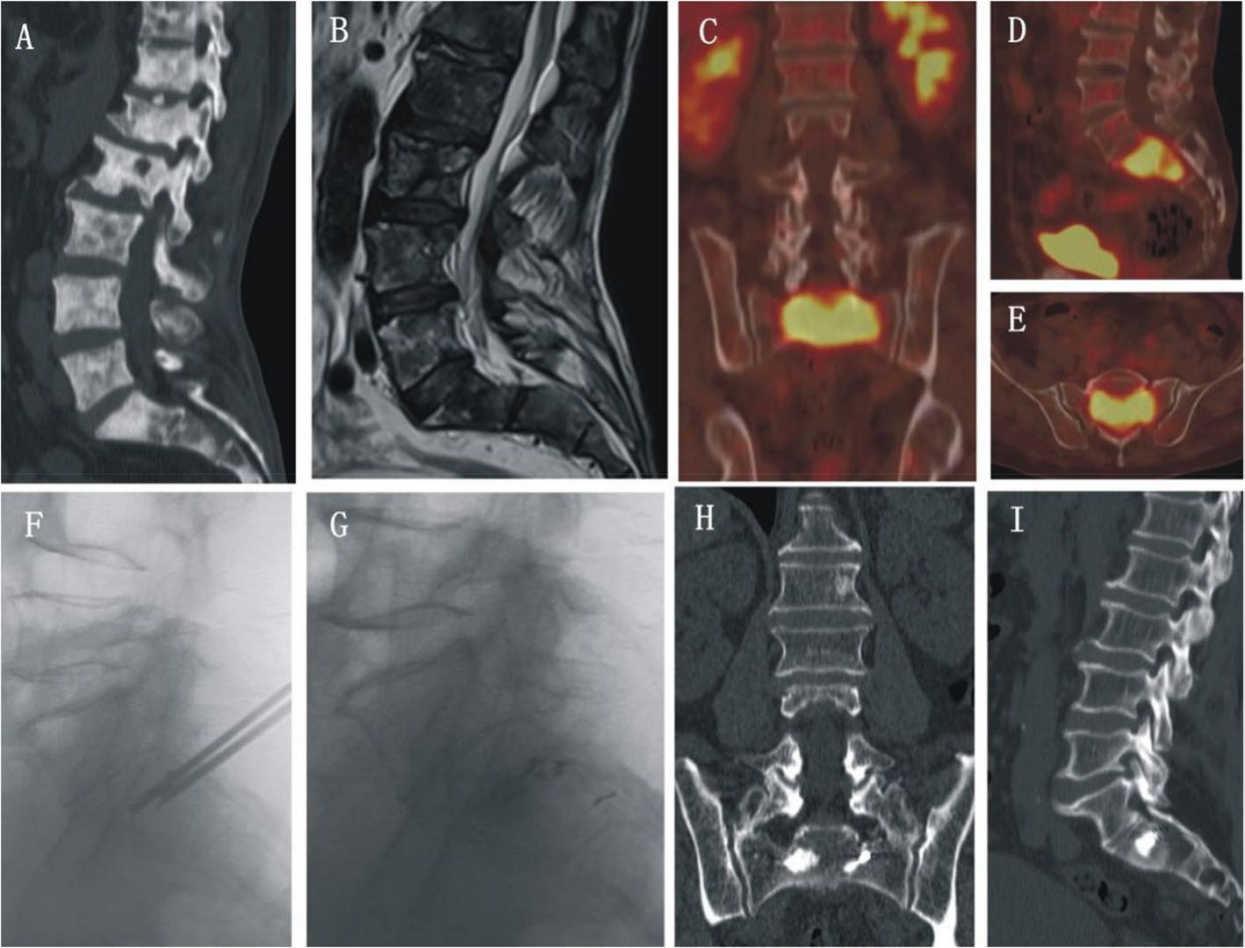
Male patient, 64 years old, with well-differentiated prostate cancer in February after combined treatment, Back pain 2 weeks preoperative VAS score of 8.7. **A**–**E** Preoperative CT, MRI, and PET-CT show sacral bone destruction and soft tissue mass formation, sacral lesions involving the sacrum, and the corresponding hole. **F**–**G** S2PVP combined with ^125^I seed implantation. **H**–**I** Postoperative CT showed bone cement and ^125^I particle distribution in the lesion area, with no leakage of bone cement; Postoperative VAS score, 2.5 points, significant pain relief

**Fig. 6:**
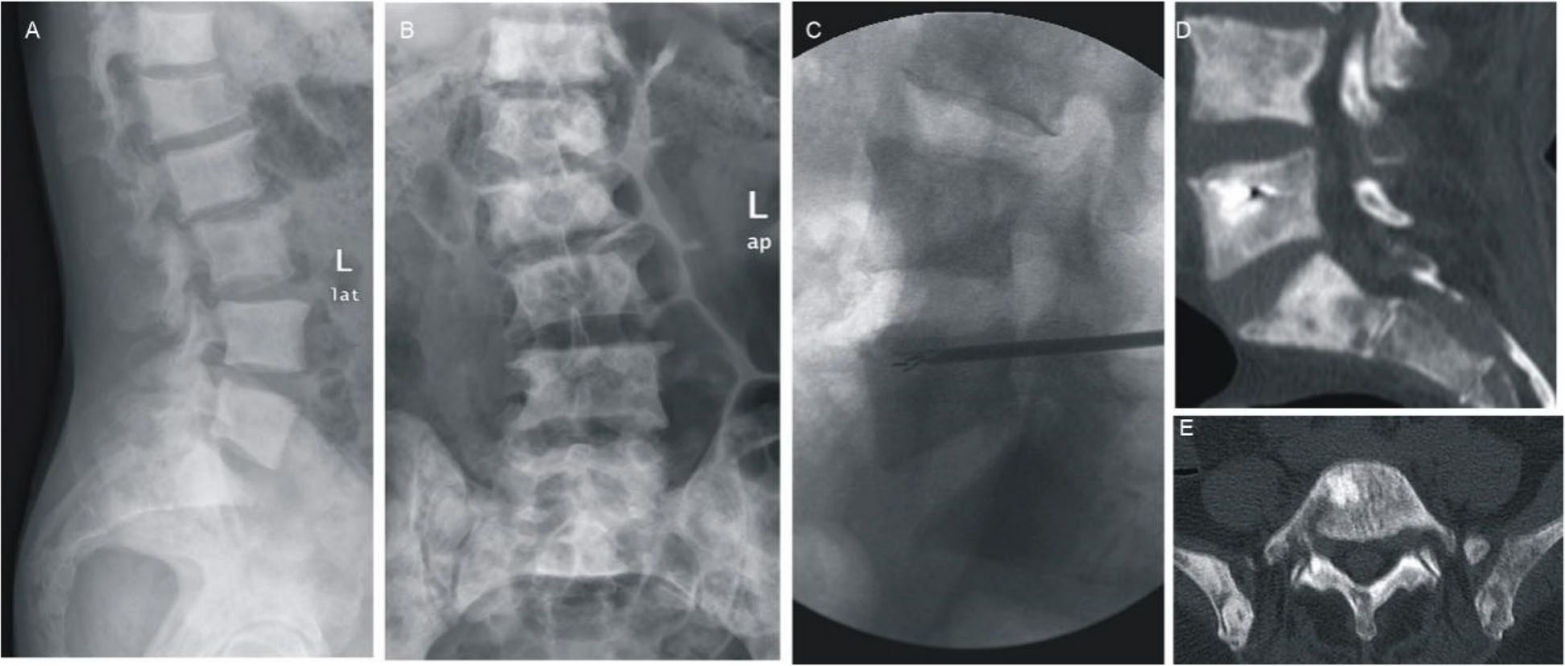
Male patient, 59 years old, well-differentiated prostate cancer after combined therapy for 1 year, back pain 8 months, preoperative VAS score of 8.5. **A**, **B** Preoperative DR shows multiple bone destruction lumbosacral, and pathological fractures. **C** L5PVP combined with ^125^I seed implantation. **D**, **E** Postoperative CT showed bone cement and ^125^I particle distribution in the lesion area, without leakage of bone cement. Postoperative VAS score 2.3 points, significant pain relief

**Fig. 7:**
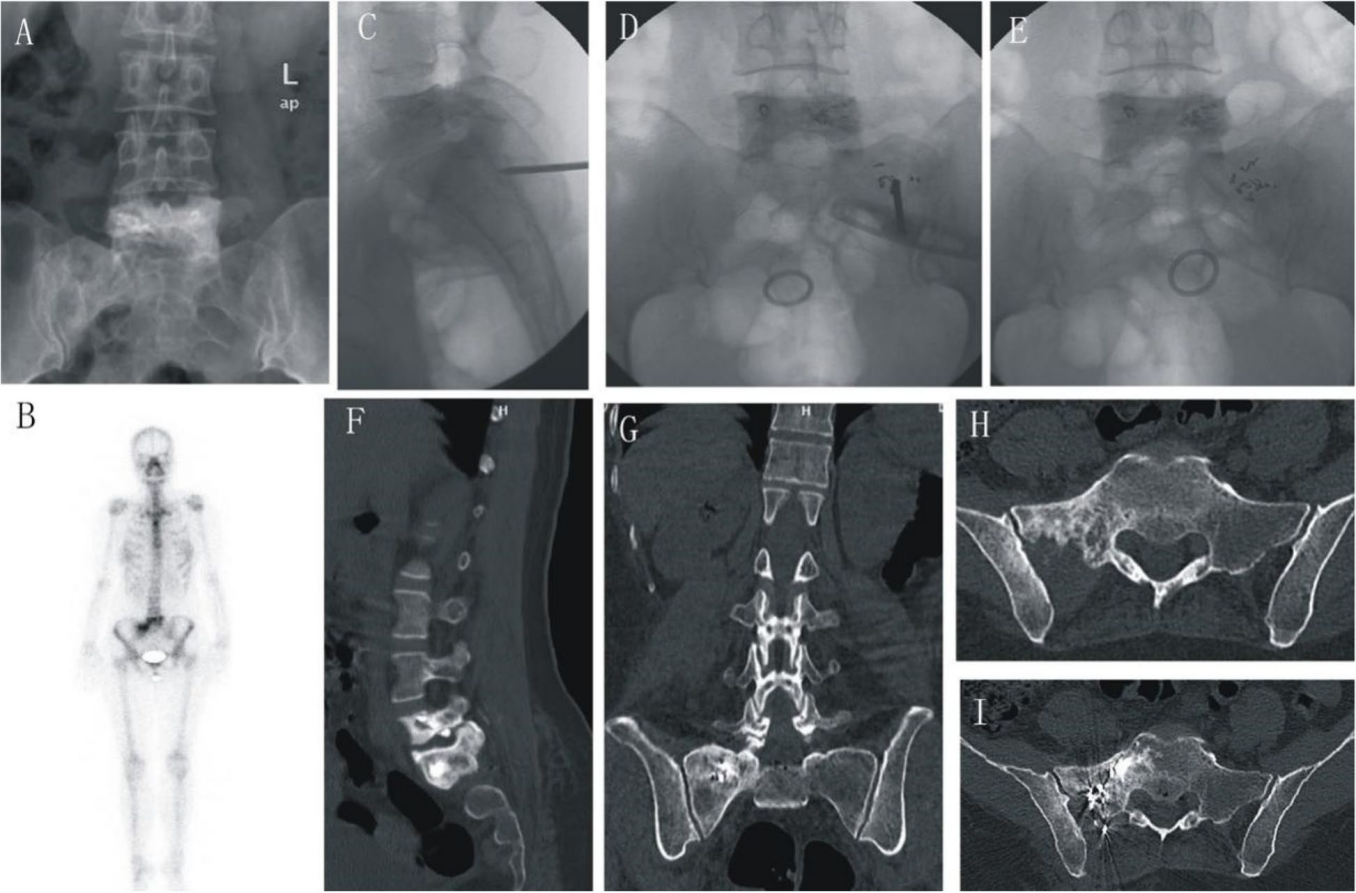
Female patients, 4 years after radical resection of the right lung, L5PVP combined with ^125^I seed 3 years after implantation, Preoperative VAS score 8.7 points. **A** Preoperative DR, CT shows that the right side of the block of S1 vertebrae and the adjacent vertebrae, and right iliac bone increased in inhomogeneity; B: SPECT: To the right sacroiliac joint abnormalities metabolically active, more consideration transferred. **C**–**E** S1PVP combined with ^125^I seed implantation. **F**–**I** Postoperative CT showed bone cement and ^125^I particle distribution in the lesion area, without leakage of bone cement. Postoperative VAS score 2.7 points, significant pain relief

The prognostic index (PI) can be calculated according to the prognostic factors determined using the Cox regression model and has important clinical significance. The larger the PI, the worse the prognosis; the smaller the PI, the better the prognosis (PI = 1.154 × age + 0.832 × primary tumor + 0.647 × spinal segment). Typical cases are shown in Figs. 5, 6, and 7.

## Discussion

Osteogenic metastatic carcinoma often presents as nodular, spotted, and diffuse multiple lesions on X-ray. Although osteogenic metastasis may manifest as irregular diffusion, the typical osteogenic metastasis is nodular and lacks the sharp shape of bone islands. The treatment methods for spinal lumbosacral osteoblastic metastases include radiotherapy, chemotherapy, surgery, and other comprehensive treatments [23, 36]. Minimally invasive surgery effectively relieves pain, preserves and restores nerve function and spinal stability, and improves quality of life.

Predicting survival and clinical treatment outcomes has always been puzzling for clinicians. Most scholars believe that survival time is related to the malignancy of the tumor, the general condition of the whole body, and metastasis to important organs [37, 38]. Treating osteoblastic metastases requires multidisciplinary cooperation, and the patient’s general state, nerve damage, pain, life expectancy, growth rate of the primary tumor, and spinal segment invasion by the tumor must be comprehensively considered before determining the surgical treatment plan and economic conditions [39].

The rational selection of surgical methods is closely related to patient prognosis. However, lumbosacral fusion might narrow the hip joint space and increase the risk of hip osteoarthritis, especially the patients with long-segment lumbosacral fusion [40]. Radiotherapy using ^125^I seeds implanted between tissues has developed rapidly in recent years and has become a typical example of the best conformal treatment [41, 42]. ^125^I seed implantation was reported in 239 patients with T1–T2 prostate cancer, and with 5-year and 10-year survival rates of 74% and 66%, respectively [43].

In this study, repeated-measures analysis of variance was used to compare the VAS scores at different time points between the combined treatment and pure PVP surgery groups. The VAS scores for the different surgical methods in the two groups changed over time, and the VAS scores on the 1st day after surgery significantly decreased. The pain of the patients was effectively relieved, while the pain in the simple treatment group was relieved 1 day and 1 month postoperation; however, the pain level gradually increased 3 months after the operation and then remained at a certain level. The pain level in the combined treatment group was significantly lower than that in the PVP group. We conducted a *t*-test on the KPS scores of the combined treatment and pure PVP groups before and 1 month after surgery. The results showed that the KPS scores of the two groups significantly increased 1 month postoperation, indicating that the physical status of all patients improved in the first month after the operation. There was a significant difference between the groups before surgery and 1 month after surgery (*p* = 0.000), and the physical status of patients in the combined treatment group was significantly better than that in the PVP treatment group. Further, univariate statistical analysis revealed that the survival rate of the PVP group was significantly lower than that of the combined treatment group (*P* = 0.038). Thus, reasonable selection of the surgical method is closely related to the prognosis of lumbosacral vertebral metastases.

This study showed that the higher the KPS score, the longer the median survival of the patients at 14 and 20 months, respectively, and the difference in survival rate was statistically significant (*P* = 0.020). Therefore, KPS is an important indicator for determining the prognosis of patients with spinal lumbosacral bone metastasis. In recent studies, the KPS has been used as an important prognostic factor in the preoperative scoring system for spinal metastases [44, 45], which is consistent with the results of this study. Thus, the preoperative KPS can be used as an important reference indicator for treating lumbosacral osteoblastic metastasis.

Percutaneous bone cement puncture osteoplasty is a minimally invasive surgical technique that enhances the local stability of the bone by injecting bone cement into the damaged area. It is used to treat bone metastases and effectively improves the quality of life of patients after surgery. The injection volume of bone cement is generally 2–9 mL, with an average of 4.5 mL for the thoracic spine and 6.0 mL for the lumbar spine. After the completion of bone cement injection, a waiting period of 15–20 min is observed to allow the polymerization reaction to complete, and then radiography and CT scans were performed for re-examination. There is no interaction between the bone cement and the radioactive iodine ions. ^125^I seed implantation has attracted widespread attention as an effective technique for interstitial brachytherapy. It involves the implantation of radioactive particles with fixed specifications and activity in the vicinity of the tumor tissue through minimally invasive methods, providing low-dose and long-term irradiation to the tumor tissue, which can improve local control and survival rates. Generally, 12–20 ^125^I particles are implanted, with an average of 16.5 particles.

In tumor epidemiological studies, researchers usually focus on comparing different therapies and identifying factors with independent prognostic value. However, little research has been conducted on patient prognostic categories and their use in choosing the best treatment strategy. A prognostic index (PI) with sufficient prognostic power can help clinicians make scientific and reasonable treatment plans for patients with tumors and largely predict long-term patient survival.

In this study, Cox multivariate regression analysis results further showed that patient age, primary tumor growth, and metastatic spinal segment were independent factors influencing the long-term survival of patients with lumbosacral osteoblastic metastases. We established a risk function equation, and in clinical practice, the PI can be calculated using the model of this study to assess prognosis. In future clinical trials, the rational grouping of patients based on individual prognostic indices may be possible. Additionally, our comparison of PVP alone and PVP combined with ^125^I seed implantation for treating patients with lumbosacral osteoblastic metastases showed that the treatment effect of PVP combined with ^125^I seed implantation was better than that of PVP alone. Univariate and Cox multivariate regression analyses confirmed that the preoperative KPS score, segment of spinal metastases, and growth rate of the primary tumor were closely related to the survival rate of patients with lumbosacral osteoblastic metastases. However, the treatment strategy for lumbosacral osteoblastic metastases requires comprehensive multidisciplinary treatment. Based on the patient’s condition, an individualized treatment plan should be formulated, incorporating the current prognostic evaluation system and comprehensive evaluation, to develop a practical and effective treatment plan. However, the reliability and accuracy of the results of this study require a large number of case summaries, scientific statistics, and large-scale prospective randomized controlled studies.

## Data Availability

The datasets used and/or analyzed during the current study are available from the corresponding author on reasonable request.
